# Preventing technique‐related complications in spinal cord stimulation trials: The Dural Substitute Confetti technique. A retrospective monocentric analysis

**DOI:** 10.1111/papr.13426

**Published:** 2024-10-22

**Authors:** Alessandro Dario, Luca Ferlendis, Bianca Bossi, Davide Locatelli

**Affiliations:** ^1^ Division of Neurosurgery, Department of Biotechnology and Life Sciences University of Insubria, Ospedale di Circolo e Fondazione Macchi Varese Italy

**Keywords:** complications, dural substitute, spinal cord stimulation, technique, trials

## Abstract

**Background:**

Spinal Cord Stimulation (SCS) is an established therapy for chronic pain, employing screening trials to identify suitable candidates before implantation. However, complications arising from both technique and medical factors present challenges to this practice. This study introduces the *Dural Substitute Confetti* technique, which addresses technique‐related complications during SCS implantation by preventing scar‐induced lead migration or breakage and reducing operating times.

**Methods:**

We conducted a retrospective analysis on 174 patients treated with SCS trials from 2017 to 2022 at our institution. Of these, 85.1% proceeded to permanent implantation. During trial surgery, synthetic dural substitutes (DS) were used to protect leads, which remained connected to an external pulse generator (EPG) for 20–28 days (mean 21.4 days). Utilizing the *DS Confetti technique*, leads were easily dissected from the DS during the second surgery and connected to an internal pulse generator (IPG). We compared complications and surgical times before and after the introduction of this technique in 2017.

**Results:**

Following the complete SCS trial, patients experienced over 50% pain relief, with an implant‐to‐trial ratio of 85.1% and a mean follow‐up of 52 months. No technique‐related complications occurred during the trial period post‐2017, while the pre‐2017 group had a 3.9% lead migration rate due to scarring, necessitating re‐implantation. The average surgery duration decreased from 54 min pre‐2017 to 32 min post‐2017. Medical‐related complications included infections (2.1%) and wound dehiscence (1.3%).

**Conclusions:**

The *DS Confetti* technique prevents scar adhesion formation during screening trials, thereby facilitating and expediting the definitive SCS implantation. Additionally, it may also reduce the risk of lead migration and iatrogenic damage, potentially lowering technique‐related complications.

## INTRODUCTION

Spinal Cord Stimulation (SCS) has become a well‐established and increasingly utilized neuromodulation therapy for managing neuropathic pain over the past two decades.^1^ Neurostimulation's pain‐relief and neuroplasticity‐inducing mechanisms are complex but involve the modulation of dorsal horn neuron activity through Aβ fiber and inhibitory interneuron activation.^2^, ^3^

Clinical conditions treatable with SCS are failed back surgery syndrome (FBSS) or Persistent Spinal Pain Syndrome type 2 (PSPS‐T2),^4^, ^5^ complex regional pain syndrome (CRPS), peripheral neuropathies (PNs), and other chronic neuropathic pain disorders.^1^, ^6^, ^7^

Since the introduction of Spinal Cord Stimulation (SCS), screening trials have been employed to identify responders before implantation of a complete system.^8^ This practice continues today, with most screening trials requiring temporary connections from the internal leads to an external pulse generator (EPG) via a percutaneous extensor.

The duration of the stimulation trial period varies. In the United States, it is reported to last between 5 and 7 days, while in Europe, these trial periods tend to be longer, ranging from a minimum of 10 days to up to 4 weeks in Belgium.^9^, ^10^ Successful trials often target a pain relief threshold exceeding 50% with stable or reduced pain medications and consistent levels of daily activity.^11^, ^12^

Over the last decade, there has been a notable surge in SCS implantation rates, reflecting an approximate 12.4% annual increase in trials and a 13% average rise in implants in the United States.^13^ However, with the escalating utilization of SCS, understanding associated complications becomes paramount for effective management, given that even rare complications can lead to significant morbidity.

The spectrum of complications linked to SCS encompasses both trial and implant phases, profoundly influencing the overall safety and success of the procedures. Historically, complication rates have ranged from 31.9% to 43%, with lead migration, infection, and fibrosis emerging as common postoperative concerns.^7^, ^14^

While much attention is directed toward complications after implantation, there is limited literature focusing on complications during the trial phase.

Technique‐related complications, such as lead migration or iatrogenic damage to the leads/electrodes/extension cables resulting from displacement or scar tissue reaction, are significant because they can compromise the SCS trial and potentially cause irreversible damage to the system. This could substantially escalate the cost and duration of the procedure,^15^, ^16^ while also subjecting the patient to additional surgical risks.^14^

In particular, technique‐related complications can be attributed to the traction of the leads due to scar tissue formation, technical errors, equipment malfunctions, and anatomical challenges. Mitigating such issues necessitates meticulous attention to detail during the implantation process, underscoring the importance of comprehensive training and procedural proficiency.

In this paper, we aim to describe our surgical technique, not previously reported in the literature, aimed at reducing technique‐related complications during the definitive implantation following a successful SCS trial.

## MATERIALS AND METHODS

This study is a retrospective analysis of 174 patients who underwent SCS screening trials between 2017 and 2022 at Ospedale di Circolo e Fondazione Macchi, University of Insubria, Varese.

Medical indications for SCS trials were PSPS‐T2 in 120 patients (69.1%), CRPS in 25 patients (14.4%), and PNs in 29 patients (16.5%). The trial lasted 20–28 days (mean 21.4) and was deemed successful if there was at least a 50% improvement in neuropathic pain as reported by the patient. The duration of the SCS trial is determined by the Diagnosis‐Related Group (DRG) system of reimbursement, with a minimum of 35 days required in our institution. Of the 174 patients undergoing SCS trials, 169 were implanted via a percutaneous approach (96.5%), while 5 underwent an open approach (laminotomy).

All patients underwent a first trial surgery via SCS percutaneous/open implantation and were connected to an external pulse generator (EPG). Synthetic dural substitutes (DS) were used in all cases to cover the distal part of the leads at the site of connection to the EPG. Each DS has a price of 135 euros, while the lead has an average price of 900 euros.

The study compared these patients with those treated during the 3 years prior to the introduction of the DS Confetti technique (2015–2017), focusing on complications and surgical times.

The mean follow‐up duration was 52 months, ranging from 15 to 84 months. Patients treated with a definitive SCS implant at the first surgery were excluded from the study.

### Surgical technique

Surgeries are performed under local anesthesia and mild sedation in cases of percutaneous approach or under general anesthesia if an open approach is required. The patient is positioned prone, and standard antibiotic prophylaxis is administered (cefazolin or vancomycin, in case of a documented allergy of cephalosporins). Whenever feasible, we prioritize the percutaneous approach due to its minimally invasive nature and its association with reduced hospitalization time.^17^, ^18^ Therefore, our focus will be on detailing this technique, which was specifically employed in 96.5% of our case series.

A Tuohy needle is carefully inserted through the skin at level T12‐L2 and into the epidural space of the spine. Through this needle, the leads are advanced into the desired location (usually D8–D10) along the spinal cord under real‐time fluoroscopy guidance. Once the percutaneous leads are in the desired place, a temporary external pulse generator is connected for an intraoperative stimulation test. When the patient reports the onset of paresthesias in the territories affected by neuropathic pain, the test is considered successful. Anchor systems are used to secure the catheters at the supra‐fascial level at the insertion point, following a small median skin incision. If the intraoperative SCS test is successful, the percutaneous leads are tunneled through the subcutaneous plane on the left/right flank and then connected to an EPG via extension leads. After ensuring proper lead placement and connection, the incision sites are closed, and the patient is monitored for 24 h to properly program the neuromodulation system and manage any postoperative complications.

After 20–30 days, if the trial yields to clinical improvement, we proceed with the implantation phase. We uncover the previous surgical scars to expose the DS Confetti (see *Focus on the procedure*), making its externalization easier. After opening the DS Confetti, we extract the extension leads and attach the electrodes to definitive extension cables or to the IPG, which is then placed internally into a subcutaneous pocket.

#### Focus on the use of Dural substitute: “The DS Confetti”

**FIGURE 1:**
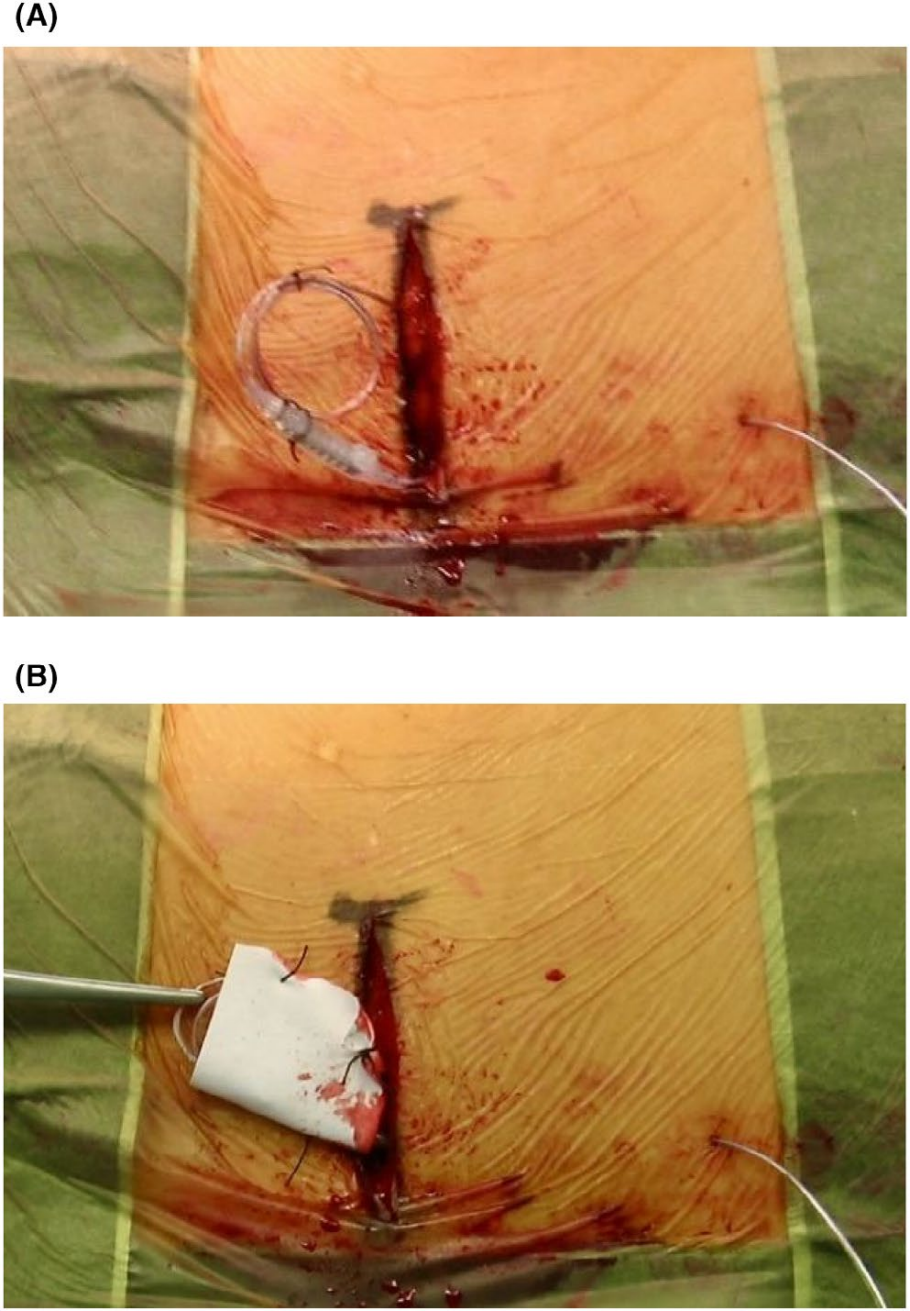
Packaging of the *DS Confetti*. (A) Step 1: Secure the catheters in a circular manner. (B) Wrap the catheters with the DS and insert the *DS Confetti* into the subcutaneous pocket.

For both percutaneous/open approaches, our surgical technique involves enveloping the terminal SCS electrodes and extension leads with a sheet of synthetic dural substitute (DS) (Neuro‐Patch©, B. Braun), forming a packaging system that we are used to calling the DS Confetti (Figure 1). The *DS Confetti* is sutured and secured in a subcutaneous pocket. This technique serves a dual purpose: first, it mitigates the risk of developing a scar tissue panel around the electrodes during the SCS trial period. This precautionary measure is essential, in addition to the anchoring system, to prevent complications that may hinder the electrodes' disentanglement and their migration.

**FIGURE 2:**
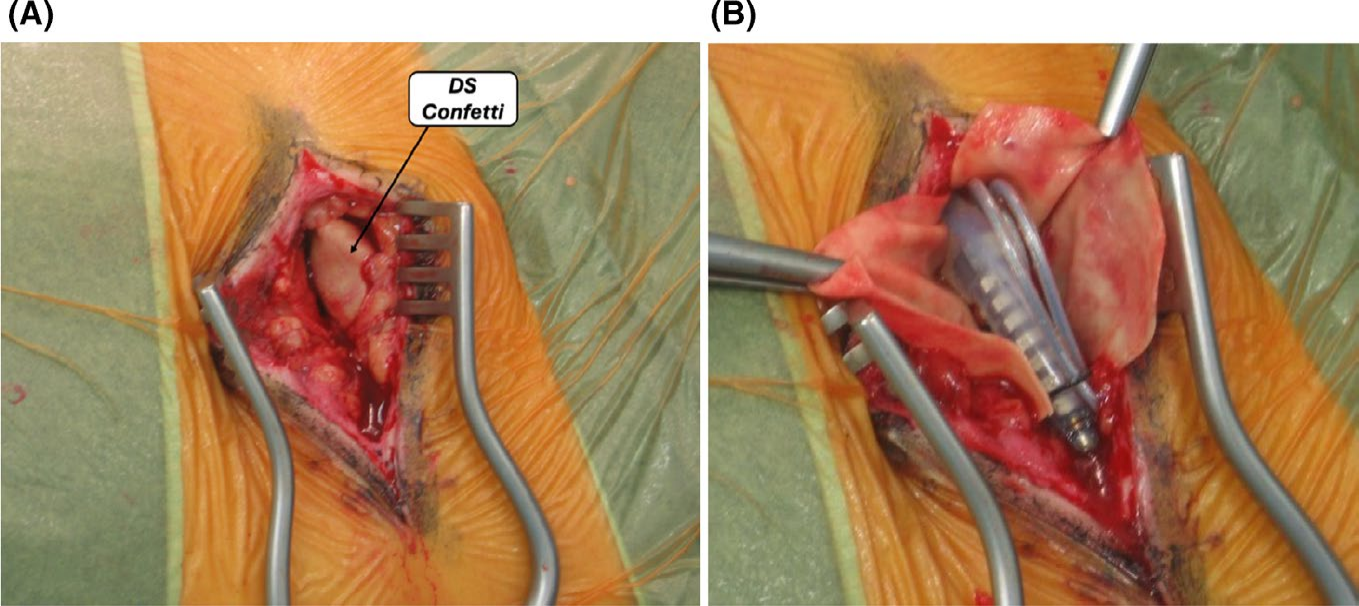
The exposition (A) and opening (B) of the DS pocket during the surgical procedure for permanent implantation of SCS.

Second, by isolating the leads from the subcutaneous tissues, the technique facilitates their dissection from the subcutaneous tissue and thus externalization during the definitive SCS surgery (Figure 2). This isolation can reduce the risk of inadvertently cutting the electrodes and enhance the overall safety and success of the surgical procedure. Finally, the easier dissection reduces the operating times of the definitive implant.

## RESULTS

The study included 174 patients, of whom 92 were females (52.9%) and 82 were males (47.1%). Among them, 148 patients had a successful trial and proceeded to permanent SCS implants, resulting in an implant‐to‐trial ratio of 85.1%. Within this group, there were 83 females (56.1%) and 65 males (43.9%). The overall mean age was 48 years.

The patients were divided into three groups for analysis: the Trial Group (TG) and the Implant Group (IG) which underwent SCS using the *DS Confetti technique*; and the non‐DS Confetti Trial Group, consisting of patients who underwent SCS trials prior to the introduction of the DS Confetti technique (from 2015 to 2017) (Table 1).

**TABLE 1:** Overview of the data from the population undergoing Spinal Cord Stimulation (SCS) implant. Persistent Spinal Pain Syndrome (PSPS‐T2); complex regional pain syndrome (CRPS); peripheral neuropathies (PNs); Male (M); Female (F).

	Trial group (TG)	Implant group (IG)	Non‐DS Confetti trial group (TG)
Total population	174 (82 M, 92 F)	148 (65 M, 83 F)	76 (34 M, 42 F)
Indication			
PSPS‐T2	120	102	36
CRPS	25	21	13
PNs	29	25	27
Complications			
Infections	4 (2.3%)	3 (2.0%)	3 (3.9%)
Wound dehiscence	–	2 (1.4%)	1 (1.3%)
Hardware malfunctions	2 (1.1%)	2 (1.3%)	2 (2.6%)
Technique‐related	–	6 (4.1%)	3 (3.9%)
Trials duration (mean)—Follow‐up (mean)	21.4 days	52 months	23.5 days

In the TG, infections were reported in 4 cases (2.3%), while in the IG, there were 3 cases (2.0%), resulting in a total infection rate of 2.1%. These infections were confined to the subcutaneous plane and required system explantation followed by antibiotic therapy. Infection rates in all groups were consistent with those reported in the literature,^7^, ^8^ indicating that the DS did not significantly increase the risk of immediate or long‐term infections.

Two cases of wound dehiscence (1.3%) were reported in the IG. Hardware malfunctions numbered 2 during the TG and 2 after the definitive implantation (IG).

We considered both lead migration and iatrogenic damage to the system as technique‐related complications. No technique‐related complications were reported in the TG. However, the IG reported 6 cases (4.1%) during follow‐up, including 5 instances of lead migration and 1 case of SCS lead breakage, all necessitating revision surgery.

In the non‐DS Confetti group, there were two cases of lead migration and one instance of iatrogenic lead damage during debridement, also requiring reimplantation.

Patients treated with the *DS Confetti* technique had a mean surgery duration for definitive implant placement of 32 min (range: 27–38 min), significantly shorter than the 54 min (range: 45–67 min) recorded for the non‐DS Confetti group (Table 2).

**TABLE 2:** Comparison of surgery duration between DS Confetti group and non‐DS Confetti group (before 2017).

Time of surgery	Non‐DS Confetti group	DS Confetti group
Trial group	72′ (range 50–85 min)	74′ (range 53–88 min)
Implant group	54′ (range 45–67 min)	32′ (range 27–38 min)

Considering that the operating room cost at our hospital is 1200 euros per hour, the time savings translate to approximately 400 euros per procedure. After accounting for the cost of the DS, the net savings per procedure amount to 265 euros, demonstrating the economic sustainability of the DS Confetti technique.

## DISCUSSION

SCS is an effective neuromodulation therapy for neuropathic pain, with a steadily increasing trend in usage in recent years.^13^ Given this trend, it is crucial to understand the complications associated with these procedures for their proper management.

In a recent meta‐analysis,^19^ the main complications reported were lead migration, hardware malfunction, insufficient stimulation, and reduced coverage, following both percutaneous and open SCS implantation procedures.

In our study, we categorized lead migration as a technique‐related complication because, based on our experience, it can occur due to catheter traction caused by the formation of scar tissue in the subcutaneous pocket. Among the technique‐related complications, we also considered potential iatrogenic damage to the catheters occurring during surgical procedures. Although rare, these occurrences may result in irreversible damage to the system, resulting in a significant escalation of both the procedural costs^1^ and the risks of morbidity.

To contribute to this understanding, this study retrospectively evaluated our institution's experience with SCS screening trials over a five‐year period (2017–2022). The mean duration of trials at our institution was 21.4 days. The duration of the SCS trial is mandated by the Diagnosis Related Group (DRG) system of reimbursement, with a minimum requirement of 20 days in our institution.

We have noted that both our implant‐to‐trial ratio results and our hardware‐related complications (ie, software malfunctions) and medical–biological complications (ie, infections, wound dehiscence) are consistent with relevant literature.^7^, ^11^, ^12^, ^19^, ^20^

Specifically, in the IG we had 6 cases (4.1%) of technique‐related complications, all due to epidural catheter migration and to a break of the SCS lead at distance. However, unlike other studies, we did not report any technique‐related complications during the trial period (TG) when compared to patients treated before the introduction of the DS Confetti technique.

Regarding the surgical approach, we tend to prefer the use of a percutaneous technique for SCS trials, in agreement with authors who argue that the open approach (laminotomy/laminectomy) is associated with longer hospital stays and increased intraoperative complications.^17^, ^18^, ^21^

In particular, this work introduces an innovative technique that holds the potential to address several challenges associated with the placement of SCS lead in subcutaneous tissues during the trial period or inadvertent catheter damage during the system's finalization procedure. The use of the *DS Confetti* protecting the leads not only aims to enhance the precision and accuracy of SCS placement but also seeks to mitigate the risks commonly associated with subcutaneous tissue manipulation. This technique can reduce the risk of lead migration due to definitive implant maneuvers, especially since the incidence of radiological lead migration appears to be around 78%.^22^

Dural substitution products are commonly employed in neurosurgery to replace the dura mater and prevent cerebrospinal fluid leakage. Synthetic dural substitute (DS) products typically consist of non‐absorbable polyester urethane (eg, Neuro‐Patch©, B. Braun). Long‐term clinical trials have demonstrated that even after many years of implantation, there is no alteration in the physical and chemical properties of these materials.^23^, ^24^ Consequently, the properties of these products, such as tissue tolerance and good suturability, ensure effective isolation of the leads from subcutaneous and scar tissues.

The *DS Confetti* technique offers two essential advantages. The first is to prevent the formation of scar adhesions around the catheters within the subcutaneous pocket. Such adhesions can potentially exert traction on the upstream system, leading to lead migration at the epidural level.

The second advantage is to facilitate the retrieval of the extension leads during the finalization procedure, reducing surgical time and potential morbidities.

The alternative use of a dural substitute may raise some concerns, but currently, dedicated synthetic materials for this procedure are not available on the market. The development of a synthetic material with properties similar to DS, potentially at a lower cost, for dedicated utilization in such procedures holds promise for future exploration.

The use of heterologous synthetic material, being a foreign body, inherently carries an increased risk of infection. However, in the TG, we reported only 4 infections (2.3%). This infection rate is lower compared to literature reports, which show infection rates ranging from 2.45% to 5% in large series and meta‐analyses and up to 14% in small series.^7^, ^8^ This data indicates that not only did we achieve a lower infection rate than those reported in other studies, but also that the use of heterologous synthetic material did not increase the overall risk of infection.

**FIGURE 3:**
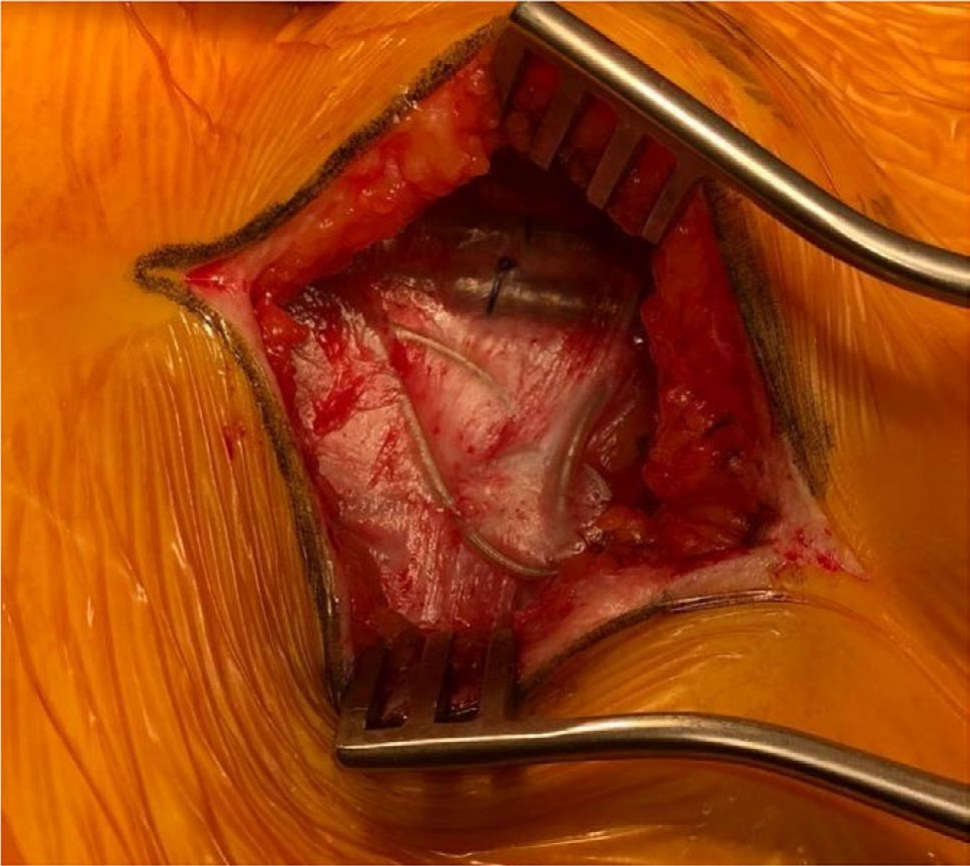
Case dating back before the introduction of the DS Confetti. Note the fibrous adhesions complicating and endangering catheter extraction.

We introduced the DS Confetti technique in 2017. Before its implementation, as shown in Figure 3, the formation of scar tissue during SCS trials posed a significant risk during catheter dissection from the surrounding tissues. In contrast, with the introduction of our technique, the leads can be easily and rapidly isolated and connected to the IPG.

In our healthcare setting, a SCS lead costs 900 euros. Repositioning a lead entails additional expenses for a new device, along with hospitalization and operating room costs. Consequently, the potential savings from preventing displacement are significant, particularly since in cases of PSPS II, we typically use two epidural leads.

The mean surgical time in the TG using the DS Confetti in the trial surgery is 74 min. By comparing the surgical times of 76 procedures conducted before 2017, we observed a similar duration for the TG percutaneous SCS surgery, with only a 2‐min saving, potentially attributable to the DS Confetti preparation in the other group. However, the time for the definitive implantation (IG) showed an average reduction of 22 min in favor of *DS Confetti* (Table 2). This reduction in surgical time is crucial for decreasing surgical morbidities and enhancing the cost‐effectiveness of the procedure.^25^ Although a 20‐min reduction might not significantly impact morbidity reduction on its own, it is important to consider that these procedures are performed under local anesthesia and mild sedation. Consequently, reducing the surgical time decreases patient discomfort and increases compliance, thereby enhancing the overall effectiveness of the surgery.

Since the time of use of the operating room has a cost, the reduction of the same in our healthcare reality can be estimated as a saving of approximately 265 euros for each procedure.

Unfortunately, the mean surgery time is often not reported in the literature, which limits our ability to directly compare our results and the potential time reduction associated with the *DS Confetti technique* to other studies. This lack of comparable data underscores the need for more comprehensive reporting of surgical times in future studies to better assess the efficiency and effectiveness of new techniques.

The duration of the trial itself poses another limitation to the study. This is because trial times vary between countries. For instance, while in Europe the trial period ranges from 10 to 28 days, in the USA it typically lasts less than a week.^9^, ^10^ This variability not only makes the reported data on trial period complications in the literature heterogeneous but also inherently limits the broader applicability of this technique. The *DS Confetti technique* is particularly suited for countries where the trial period is approximately longer than 10 days, as this allows sufficient time for scar tissue reactions to potentially affect subsequent implantation surgery.

Another primary limitation of this study lies in its monocentric design. While this approach enhances the study's generalizability, it introduces variability in procedural techniques and patient demographics. To ascertain the broader applicability of the proposed technique and its risk reduction benefits, further research across different medical centers is warranted. Future studies involving diverse patient populations will provide a more comprehensive assessment of the technique's effectiveness in reducing complications associated with subcutaneous tissue manipulation and catheter damage.

## CONCLUSION

The management of complications in SCS surgery continues to pose a significant challenge. The implementation of the *DS Confetti* technique offers significant advantages in mitigating complications and optimizing the implantation process for the definitive SCS system. By preventing the formation of scar adhesions, this technique streamlines the transition to the complete system, enhancing efficiency and patient outcomes. Moreover, the reduced risk of lead migration and iatrogenic damage during SCS trials contributes to a substantial decrease in technique‐related complications. While further clinical studies and validation are warranted to establish the efficacy and safety profile of this innovative technique, its introduction represents a step forward in addressing critical challenges associated with SCS. The potential benefits of this method could lead to improved patient outcomes, reduced complications, and increased overall success rates in SCS procedures. Furthermore, studies aimed at developing material similar to DS for specific use in SCS trials could be beneficial.

## AUTHOR CONTRIBUTIONS

Alessandro Dario: Conceptualization, Surgeon, Supervision; Luca Ferlendis: Conceptualization, Writing—Reviewing and Editing; Bianca Bossi: Writing, Reviewing and Editing, Data Collection; Davide Locatelli: Supervision.

## CONFLICT OF INTEREST STATEMENT

The authors have no personal, financial, or institutional interest in any of the drugs, materials, or devices described in this article.

## INFORMED CONSENT

The participants and any identifiable individuals consented to publication of his/her image.

## Data Availability

The data that support the findings of this study are available on request from the corresponding author. The data are not publicly available due to privacy or ethical restrictions.
